# Weight loss referrals for adults in primary care (WRAP): protocol for a multi-centre randomised controlled trial comparing the clinical and cost-effectiveness of primary care referral to a commercial weight loss provider for 12 weeks, referral for 52 weeks, and a brief self-help intervention [ISRCTN82857232]

**DOI:** 10.1186/1471-2458-14-620

**Published:** 2014-06-18

**Authors:** Amy L Ahern, Paul N Aveyard, Jason CG Halford, Adrian Mander, Lynne Cresswell, Simon R Cohn, Marc Suhrcke, Tim Marsh, Ann M Thomson, Susan A Jebb

**Affiliations:** 1 MRC Human Nutrition Research, Elsie Widdowson Laboratory, Fulbourn Road, Cambridge CB1 9NL, UK; 2 Nuffield Department of Primary Care Health Sciences, University of Oxford, Radcliffe Observatory Quarter, Woodstock Road, Oxford OX2 6GG, UK; 3 Department of Psychological Sciences, University of Liverpool, Eleanor Rathbone Building, Bedford Street South, Liverpool L69 7ZA, UK; 4 MRC Biostatistics Unit Hub for Trials Methodology, Institute of Public Health, University of Cambridge, Forvie Site, Robinson Way, Cambridge CB2 0SR, UK; 5 Else Kröner-Fresenius-Center for Nutritional Medicine, Technische Universität München, Munich, Germany; 6 The Primary Care Unit, Institute of Public Health, University of Cambridge, Forvie Site, Robinson Way, Cambridge CB2 0SR, UK; 7 Faculty of Medicine and Health Sciences, University of East Anglia, Norwich NR4 7TJ, UK; 8 UK Health Forum, Fleetbank House, 2-6 Salisbury Square, London EC4Y 8JX, UK; 9 Department of Health Services Research and Policy, London School of Hygiene and Tropical Medicine, London WC1H 9SH, UK; 10 Centre for Health Economics, University of York, York YO10 5DD, UK

**Keywords:** Obesity, Weight-loss, Primary care, Adults

## Abstract

**Background:**

Recent trials demonstrate the acceptability and short term efficacy of primary care referral to a commercial weight loss provider for weight management. Commissioners now need information on the optimal duration of intervention and the longer term outcomes and cost effectiveness of such treatment to give best value for money.

**Methods/Design:**

This multicentre, randomised controlled trial with a parallel design will recruit 1200 overweight adults (BMI ≥28 kg/m2) through their primary care provider. They will be randomised in a 2:5:5 allocation to: Brief Intervention, Commercial Programme for 12 weeks, or Commercial Programme for 52 weeks. Participants will be followed up for two years, with assessments at 0, 3, 12 and 24 months. The sequential primary research questions are whether the CP interventions achieve significantly greater weight loss from baseline to 12 months than BI, and whether CP52 achieves significantly greater weight loss from baseline to 12 months than CP12. The primary outcomes will be an intention to treat analysis of between treatment differences in body weight at 12 months. Clinical effectiveness will be also be assessed by measures of weight, fat mass, and blood pressure at each time point and biochemical risk factors at 12 months. Self-report questionnaires will collect data on psychosocial factors associated with adherence, weight-loss and weight-loss maintenance. A within-trial and long-term cost-effectiveness analysis will be conducted from an NHS perspective. Qualitative methods will be used to examine the participant experience.

**Discussion:**

The current trial compares the clinical and cost effectiveness of referral to a commercial provider with a brief intervention. This trial will specifically examine whether providing longer weight-loss treatment without altering content or intensity (12 months commercial referral vs. 12 weeks) leads to greater weight loss at one year and is sustained at 2 years. It will also evaluate the relative cost-effectiveness of the three interventions. This study has direct implications for primary care practice in the UK and will provide important information to inform the decisions of practitioners and commissioners about service provision.

**Trial Registration:**

Current Controlled Trials ISRCTN82857232. Date registered: 15/10/2012.

## Background

Obesity has trebled since the 1980s and globally, excess weight is estimated to account for 44% of diabetes, 23% of ischemic heart disease and 7-41% of some cancers [1]. There is good evidence that intensive lifestyle interventions can produce weight loss linked to clinically significant health benefits [2], but such specialist interventions are costly given the high prevalence of obesity. Interventions delivered in primary care can also be demanding in terms of staff resources, set up and training and participant weight loss is often less than 5% of initial weight [3,4]. In the UK, NICE recommends consideration of any intervention that meets best practice, including referral to commercial weight loss programmes [5]. Commercial programmes are usually delivered in large groups by lay people, and preliminary evidence suggests they may be more affordable than interventions led by health professionals, making weight loss initiatives available for more individuals [6,7].

A number of commercial weight loss providers currently operate referral schemes for Public Health and the National Health Service (NHS) in the UK, whereby commissioners can purchase 12 week referral packages at a reduced cost, which are provided at no cost to patients. Two randomised controlled trials conducted by members of the current research team have demonstrated the effectiveness of commercial referrals. Jolly et al. compared a number of 12 week weight loss interventions in Birmingham’s Lighten Up service, including three commercial providers, to a control intervention (12 vouchers to attend a leisure centre) [8]. Twelve-month weight loss was significantly greater among participants referred to a commercial programme (Weight Watchers; WW) than control participants [−4.35 ± 6.9 kg vs −1.63 ± 6.0 kg; p < 0.001]. Jebb et al. [9] demonstrated that overweight and obese adults referred to this commercial programme by their primary care provider for 12 months lost twice as much weight as those who received standard care [−5.1 ± 6.1 kg vs 2.3 ± 4.2 kg; p < 0.001]. These findings suggest that referral to a commercial programme (CP) by a primary care provider is a clinically effective weight loss intervention over a one year period. However, limited data on participants who agreed to attend further follow up suggests significant weight regain beyond programme end [10].

The NHS currently provides 12 week referrals to commercial programmes. There is conflicting evidence on whether providing longer treatment interventions could result in greater and more sustained weight loss. In one meta-analysis of studies providing ‘extended care’ , participants receiving extended care had, on average, 3.2 kg less weight regain than controls over a mean follow up period of 17.6 months [11]. The reduced weight regain in the extended care intervention in studies with 6–12 month follow-up was at least 1.5 kg. However, in a recent review of behavioural weight management programmes, meta-regression of trials with longer and shorter programmes found no benefit of longer programmes up to 1 year [12]. Indirect comparisons from Jebb et al. and Jolly et al. suggest that 12 months CP (weight loss 5.1 kg) achieves greater loss than 12 weeks CP (weight loss 4.4 kg, assessed at 12 months). The difference is small, but participants in Jolly at al. were heavier and older than those in the Jebb et al., two factors associated with greater weight loss in an audit of the CP’s NHS referral database [13] and an observational analysis of the routine Lighten Up service [14]. Thus we might anticipate the difference in weight loss after 12 or 52 weeks intervention in comparable groups to be greater than the comparison between these two studies. Moreover, further analysis of Jolly et al. suggests the apparent impact of the WW intervention may have been atypically high. In the two other commercial providers (Slimming World and Rosemary Conley), mean weight loss at 12 months was smaller than WW, yet a much larger comparison (n = 3000) of the three providers in the routine Lighten-Up referral service shows that mean self-reported weight loss at 1 year in those attending WW was very close to the mean weight loss across all providers [14]. Mean 12 month weight loss for the three CPs in Jolly et al. was 2.7 kg, giving an assumed difference of 1.36 kg between this and the 52 weeks intervention in Jebb et al. A formal RCT is needed to show whether the greater loss in the 12 month programme is due to the longer referral and the current trial will directly compare weight loss at 12 months for participants receiving 12 weeks referral (CP12) and 52 weeks referral (CP52).

Obesity is a chronic, relapsing condition and the sustainability of weight loss achieved in short term interventions cannot be assumed. There is currently no published data on 12 week commercial referral outcomes beyond 12 months. In the limited data from participants from Jebb et al., 12 months CP did lead to greater weight loss than standard care, but this difference was small and sensitive to assumptions about missing data [10]. We will therefore follow participants up for 24 months to examine whether any initial differences in weight loss are sustained in the longer term.

Careful consideration has been given to the most appropriate control intervention. Since in many cases, obesity remains untreated in primary care, a no-intervention control may be considered to reflect standard care. However, recognition of obesity by GPs as part of recruitment to the trial and appointments for outcome measurements may constitute an intervention in its own right and in a recent review even minimal intervention ‘control’ groups lost weight [12]. Where offered, weight management interventions in primary care vary considerably. Since this is not the focus of this trial it is important to have a standardised ‘control’ intervention. Inclusion of a brief intervention based on written self-help materials will allow us to control for the impact of the GP offering a weight loss intervention and trial participation on weight loss and allow some consideration of the relative contribution of engagement and follow-up versus the nature and content of the specific intervention provided.

For NHS commissioners, one of the most important questions is whether an intervention offers value for money and a rigorous evaluation of cost-effectiveness has been built into the trial. Data on treatment costs, health-care usage and quality of life [15] will enable us to model whether any additional weight loss achieved through the 52 week programme is worth the additional costs. Initially this will consider cost-effectiveness from the perspective of the NHS, within the period of the trial (i.e. 24 months), However, ultimately, we want to know whether the interventions are likely to lead to an increase in length and quality of life, and at what cost. It is not practical to conduct a prospective study with lifetime follow up to establish this. Instead we propose using a well-developed decision-analytic model to estimate the long term impact of weight loss on risks of chronic disease and hence quality adjusted life years (QALYs) and cost.

Qualitative data suggests neither participants nor practitioners view weight management services as a priority in primary care and that some resist the idea that it is a medical issue in and of itself [16]. Thus, by delivering the intervention outside of a medical context, a CP fits better with participants’ own view of weight management. This study will examine participant experience in greater depth to explore the ways in which individuals understand and make sense of the imperative to lose weight, and the values and tensions arising from the primary care- commercial provider relationship. It will also examine the extent to which the weekly weigh in and the sense of peer support are experienced to be key aspects of the CP and the extent to which these are felt to facilitate weight loss.

Interventions for weight management could potentially be improved by developing a greater understanding of the psychosocial factors that explain individual variation in adherence, weight loss and post-intervention weight maintenance. There is a particular lack of knowledge about how these factors change during weight loss and how they affect weight maintenance. The current study will use validated questionnaires to explore a number of psychosocial factors that have either demonstrated an association with attrition, weight loss, and maintenance of weight lost in previous studies, or represent constructs identified as potentially important predictors of weight loss maintenance in recent reviews [17-19]. We will examine how baseline differences in these factors affect weight trajectories, how these factors change during and following a weight loss intervention, and how changes are associated with changes in weight.

### Objectives

#### Primary objectives

The primary research question is whether the CP interventions achieve significantly greater weight loss from baseline to 12 months than BI, and whether CP52 achieves significantly greater weight loss from baseline to 12 months than CP12.

### Secondary objectives

#### Clinical effectiveness

We will examine differences between the three interventions in weight, waist circumference, body composition, and blood pressure at 3, 12 and 24 months and differences in biochemical measures (blood glucose, total cholesterol, HDL cholesterol, LDL cholesterol, and HbA1c) at 12 months. Specifically we will test the following hypotheses:

i) Both CP interventions achieve significantly greater weight loss than BI from baseline to 3 months and baseline to 24 months and CP52 produces significantly greater weight loss than CP12 from baseline to 24 months.

ii) Both CP interventions achieve significantly greater improvements in waist circumference, body composition and blood pressure than BI between baseline and 3, 12 and 24 months.

iii) CP52 achieves significantly greater improvements in waist circumference, body composition and blood pressure than CP12 between baseline and 3, 12 and 24 months.

iv) Both CP interventions achieve significantly greater improvements in biochemical measures than BI between baseline and 12 months, and CP52 achieves significantly greater improvements than CP12.

#### Cost-effectiveness

We will examine the cost-effectiveness of each of these interventions. The following hypotheses will be tested:

i. CP52 is more cost-effective than CP12, as assessed by both within trial cost effectiveness and long term cost-effectiveness analyses.

ii. Both CP12 and CP52 are more cost-effective than BI.

### Participant experience

A qualitative workstream will explore the attitudes of participants to primary care referrals to commercial providers for weight loss, and also their wider experiences of weight management. In line with a qualitative research methodology, the following three areas will act as a guide for the research that will also remain sensitive to the experiences and topics raised by participants:

i) The extent to which participants feel that they have been referred for weight management in the NHS by their GP, and how this relates to their experience of participating in the programme and their attitudes toward weight loss.

ii) The extent to which the weekly weigh in and the sense of peer support are experienced to be key aspects of the CP

iii) The extent to which being ‘overweight’ or ‘obese’ is considered a medical issue by participants

### Psychosocial factors

This study will also examine psychosocial factors that are associated with completion of the intervention, weight loss and weight loss maintenance, to enable greater understanding of who benefits from these interventions and to inform development of new interventions.

### Biological sampling

This study will collect blood samples in order to examine changes in markers of risk of CVD and diabetes (fasting lipids, glucose and glycosylated haemoglobin). DNA will be collected for subsequent analyses of how genetic variation effects response to the interventions.

## Method

### Trial design

**Figure 1:**
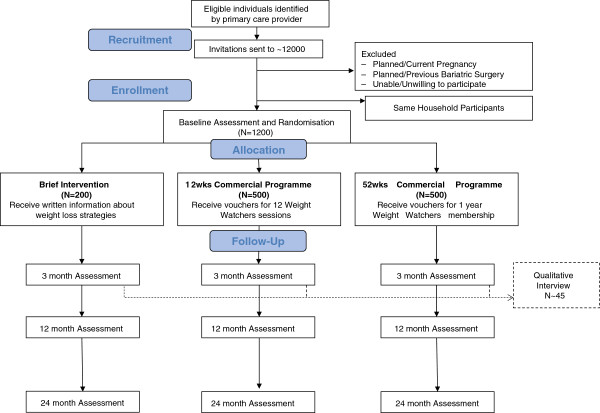
Participant flow diagram.

This is a multicentre, randomised controlled trial with a parallel design. Participants will be randomised to one of three interventions: Brief Intervention (BI), Commercial Programme for 12 weeks (CP12) or Commercial Programme for 52 weeks (CP52) in an allocation of 2:5:5 (Figure 1).

### Population

Overweight and obese adults (BMI ≥ 28) in the UK, deemed eligible for weight management intervention by their general practitioner.

### Setting

Participants will be recruited through primary care practices across England by three research centres. MRC Human Nutrition Research is the coordinating centre. They will recruit through local practices in Cambridgeshire and all measurements will be conducted by trained research staff at the research centre. The University of Liverpool will recruit through local practices across Merseyside and all measurements will be conducted by trained research staff at the research centre. The University of Oxford will recruit through practices across England and measurements will be conducted by trained health professionals (usually a research nurse) in the practice. Recruitment started in October 2012 and was completed in February 2014.

### Participants

Participants will be 1200 overweight and obese adults in England, recruited by their local primary care provider.

### Inclusion criteria

The inclusion criteria are BMI ≥28 kg/m2, aged ≥18 years, and willing and able to comply with the study procedures. For simplicity, we will not vary the BMI criteria by ethnic group.

### Exclusion criteria

The exclusion criteria are: planned or current pregnancy in the next two years; previous or planned bariatric surgery; currently following a weight-loss programme (defined as a structured, prescribed and monitored programme and not a self-regulated diet); non-English Speaking or with Special Communication needs that would preclude them from understanding the study materials and interventions. GP’s will exclude patients who are inappropriate to invite into the study, for example patients who are violent/terminally ill/have a history of an eating disorder. GPs will also be allowed to define any additional inclusion/exclusion criteria to meet local practice and will be asked to provide details on these for the reporting of the study. No further criteria will be imposed, thus capturing the population that would typically be referred to these treatments. Participants receiving other weight loss treatments, e.g. Orlistat, will not be excluded as such participants would still be eligible for commercial referrals in standard practice, but this will be adjusted for in the analyses. Participants will be randomised to intervention arms, and thus those receiving additional treatment should be evenly spread across the interventions and these treatments will be accounted for in the cost-effectiveness analyses.

### Inclusion of same household partners

Where more than one individual from a household is eligible and wants to enrol in the study, both members of the household will be allocated to the same treatment group (randomising participants at the household rather than the individual level) but only one person per household (the first to enrol) will be enrolled as a ‘participant’ who will provide measurements for the trial and attend follow-up visits. The ‘non-participant’ member(s) of a household will be referred to as ‘same household partner’, and will attend a ‘baseline’ visit to give consent and to receive their intervention materials. The ‘same household partner’ will be asked for consent to obtain their attendance and weight data from Weight Watchers (if they are allocated to this arm) through their WW NHS Referral Database. There is also potential for participants to be part of a household where other members are engaged in weight loss programmes, outside of this study. Therefore, all participants will be asked to provide information about weight loss activities within their household, regardless of whether they have a partner in the study or not.

### Recruitment

GP practices will be identified and recruited by the local Primary Care Research Network (PCRN). Practices will be targeted that do not have an existing contract with commercial weight loss services. In this way participants allocated to the brief intervention will not be denied standard care.

Based on the 10% recruitment rate from Jolly et al., we will approach approximately 12000 eligible individuals to recruit 1200 participants. The primary care provider will search their electronic registers for eligible individuals and GPs will screen out those to whom it would be inappropriate to send a letter (for example patients known to have a history of eating disorders or to be terminally ill). The letter will not mention the participant’s weight, but offers the availability of weight management and also will give brief details of the trial. Interested participants will be asked to telephone (on a designated Freephone number) or email the study co-ordinator at their local site for further information. A member of the research team will then describe the trial to the potential participant, undertake further screening, and, if agreeable, offer an appointment for baseline assessment and enrolment in the trial. This will be confirmed by letter, accompanied by a participant information sheet.

We will monitor uptake of the trial by ethnic group and by gender. GPs will be asked in their search for eligible participants to report summary statistics of the gender and ethnic composition of the eligible population. By comparing the recruited population to the eligible population we will be able to examine whether take-up of referral differed by ethnicity or gender.

### Randomisation

At the first assessment, a member of the research team trained in taking informed consent will ensure that the participant understands the trial and has read the participant information sheet. They will confirm their eligibility for the study and obtain written consent for their participation in the trial. Participant details, including baseline weight, will be entered into an online database by a member of the research team.

The database will automatically assign participants with a valid recorded baseline weight to one of three interventions (BI, CP12, CP52). The randomisation sequence was generated by the trial statistician and allocates participants in a 2:5:5 allocation stratified by centre and gender, with a block size of 12. The sequence is unknown to research staff and participants.

Due to the nature of the intervention and the trial design, neither participants nor research staff will be blinded to the intervention allocation.

### Withdrawal

Participants are free to withdraw from the trial at any time, without this affecting their care, by informing a member of the research team. Participants who withdraw will not be replaced, and data already collected will be used unless the participant requests that it be removed.

Participants might choose not to attend the commercial weight loss programme, or may stop attending sessions during the trial. Participants who withdraw from the intervention will be followed up at assessment appointments in the same way as other participants unless they also choose to withdraw from the trial.

Three contact attempts (by different means and at different times) will be made for each follow up appointment. On the third attempt to schedule an appointment, or where a participant informs us that they are unable or unwilling to attend a follow up appointment, a self-measured weight will be requested. These data will not be included in the primary analyses but will provide additional data that can be used for sensitivity analyses where it is considered appropriate.

### Interventions

#### Referral to a commercial provider

Participants who are assigned to the two commercial referral arms will receive vouchers to attend Weight Watchers sessions and asked to attend a local meeting that is convenient for them. They will be asked not to mention their participation in the trial to the group leader or other members, to make their experience as representative as possible.

**CP12:** Participants allocated to the 12 week referral will receive free vouchers to attend 12 Weight Watchers sessions and access to their internet resources for 16 weeks. This is the package currently used in the WW NHS Referral Scheme and currently costs the NHS £55 + VAT.

**CP52:** Participants allocated to the 52 week referral will receive free vouchers to attend 52 sessions of Weight Watchers and access to their internet resources for 12 months. This packages is estimated to cost the NHS £190 + VAT.

### Brief intervention

The control intervention is a standardised brief intervention: recognition of the problem by the GP (letter of invitation), basic written information on self-help weight loss strategies provided by a member of the research team at the baseline visit (British Heart Foundation Booklet: So you want to lose weight… for good) and weighing at follow up (coincides with outcome measurements at 3, 12 and 24 months).

### Adherence

Attendance at CP meetings will be monitored both through self-report at assessment appointments and data collected by WW at weekly meetings (which can be provided, with consent, through the WW NHS referral database and tracked using NHS referral ID) and these data will be controlled for in sensitivity analyses. Similar information may be available from WW regarding website usage, and this data will be combined with that collected via self-report. We will also collect self-report data on the extent to which BI participants have used their self-help materials.

### Outcomes

#### Clinical effectiveness outcomes

The primary outcome will be body weight (kg) at 12 months. Secondary clinical outcomes will be: body weight (kg) at 3 and 24 months, whether a participant has lost ≥5% and ≥10% of initial body weight at 3, 12 and 24 months; waist circumference, body composition, and blood pressure at 3, 12 and 24 months; blood glucose, total cholesterol, HDL cholesterol, LDL cholesterol, and HbA1c at 12 months.

### Cost-effectiveness outcomes

The incremental cost-effectiveness ratio (ICER) of the intervention is the main outcome of the economic evaluation and will be expressed as incremental costs per incremental change in weight/BMI for the within-trial evaluation.

### Adverse events

This is a low risk trial with little reason to consider that adverse events would arise as a result of following any one of the interventions. Accordingly no formal adverse event monitoring is planned.

### Visits and measurements

Participants will attend measurement appointments at 0, 3, 12 and 24 months. Details of which measures will be taken at which appointments are summarised in Table 1.

**Table 1:** Schedule of enrolment, interventions, and assessments

	**STUDY PERIOD**				
	**Enrollment**	**Baseline visit**	**Post allocation**		**Close-out**
**TIMEPOINT**	***-t***_***1***_	**0**	**3 months**	**12 months**	**24 months**
**ENROLMENT:**					
**Eligibility screen**	X	X			
**Informed consent**		X			
**Allocation**		X			
**INTERVENTIONS:**					
***Brief Intervention***		X			
***12 weeks Commercial Programme***		X	X		
***52 weeks Commercial Programme***		X	X	X	
**ASSESSMENTS:**					
***Height***		X			
***Weight***		X	X	X	X
***Fat mass***		X	X	X	X
***Waist Circumference***		X	X	X	X
***Blood Pressure***		X	X	X	X
***Blood Glucose***		X		X	
***Lipid Profile***		X		X	
***HbA1c***		X		X	
***DNA***		X			
***Demographics Questionnaire***		X	X	X	X
***Health Care Usage Questionnaire***		X	X	X	X
***EQ5D***		X	X	X	X
***Psychosocial Questionnaires***		X	X	X	X
***Intervention Usage Questionnaires***		X	X	X	X
***Qualitative Interviews (subset only)***			X		

### Clinical measurements

All clinical measurements will be made in line with standardised operating procedures by trained research staff. Participants will be asked to remove shoes and heavy clothing items. Height (cm) will be measured in cm using a stadiometer. Weight and fat mass will be measured in kg using a Tanita segmental body composition analyser. Waist circumference (cm) will be measured using a tape measure, half way between the lowest rib and the iliac crest. Blood pressure will be measured using standardised methods.

### Biochemical measurements

Biochemical measurements are optional for participants and taken under separate consent. Blood samples will be taken by fully trained research staff in line with standardised operating procedures. For assessment appointments where blood will be taken (0 and 12 months), participants will be asked to attend in a fasted state (no food or drink for 12 hours prior to the appointment).

At baseline and 12 months, participants will provide a sample of whole blood for analysis of glucose, glycosylated haemoglobin (HbA1c) and lipid profile. At baseline, an additional sample of whole blood will be collected in an EDTA tube, frozen at −80 and stored for later extraction of DNA and outside the trial protocol.

### Health care usage measures

At each visit, participants will complete a Health Care Usage Questionnaire to assess their use of Health Care Services in the last 3 months.

### Psychosocial measures

Participants will complete a series of questionnaires at or before each assessment to assess psychosocial factors related to weight control.

*The Flexible and Rigid subscales if the Eating Inventory*[20] measure cognitive dietary restraint and distinguish flexible and rigid dietary restraint strategies.

The *Power of Food Scale (PFS)*[21] measures individual differences in hedonic hunger (hunger in the absence of energy need).

A visual analogue scale (VAS) will be used to assess state hunger at the time the questionnaire is completed [22]. Sensitivity analyses can examine whether this influences responses to questionnaires about eating behaviour.

*The Self-Report Habit Index (SRHI)*[23] is a measure of behavioural frequency, automaticity and identity, which has also been used to measure the automaticity of thoughts, such as body-related cognitions. In the current study, we will use this measure to examine the extent to which “watching what I eat” and “exercising regularly” become automatic and are seen as part of a participant’s identity, and the extent to which this predicts weight loss and weight loss maintenance.

*The Treatment Self-Regulation Questionnaire; Diet Self-Regulation Questionnaire (DSRQ) and Exercise Self-Regulation Questionnaire (ESRQ)* measure the extent to which a participant’s motivation to participate in treatment, eat a healthy diet, or exercise, is autonomous (i.e. they are motivated by personal reasons) or controlled (i.e. they are motivated by perceived pressure from others).

*The Problem Eating Behaviours Questionnaire (PEBQ)*[24] measures the extent to which particular eating behaviours are problematic for participants.

The *EQ5D*[15] is a self-report measure of quality of life, which will be used to calculated QALYs for the cost-effectiveness analysis.

We will measure life satisfaction using the *Satisfaction with Life Questionnaire (SLQ)*[25] and depression and anxiety using the *Hospital Anxiety and Depression Scale (HADS)*[26]*.*

### Qualitative data collection

A subset of participants from the Cambridge centre will be recruited to participate in a qualitative study. Data will be collected through semi-structured interviews with up to 15 participants in each intervention. A maximum-variation (heterogeneity) sampling technique will be used to select potential interviewees based on demographic information obtained during the telephone screening questionnaire and through a questionnaire at the baseline visit. At the 3 month visit selected participants (including some who have dropped out of treatment but not withdrawn from follow-up) will be invited to participate in an interview. Participants will be offered the choice between having the interview at their home or in a private office at the University of Cambridge. Interviews will not be held where study procedures are conducted, to reduce associations between the interview and the measurement visits of the trial in order to encourage participant’s to speak openly about their experiences of the intervention to which they have been assigned. Interviews will last approximately one hour and will follow a general topic guide that will be piloted with a subset.

### Statistical analysis

#### Analysis design

There is already good evidence to suggest that CP produces significantly greater weight loss than BI and in the event that CP is not better than BI then the comparison of the CP arms would not be of interest. Accordingly we will conduct a sequential analysis, which will preserve the Type 1 error of 5% without the need for a multiplicity correction such as Bonferroni. The sequential analysis will consist of the following 2 stages:

i) Test the one-sided hypothesis that weight loss in the CP groups combined is greater than the weight loss in the BI arm.

ii) If the first test is significant at the 5% significance level, then test the two-sided hypothesis that there is a difference between CP52 and CP12 weight loss at the 5% significance level.

### Sample size calculation

We based the power calculation on data from our previous trials [8,9] with an expected difference of 2.3 kg between BI and combined CP, 1.3 kg difference between CP12 and CP52 (for example, a weight loss of 1.05 kg in the BI arm, 2.7 kg in the CP12 arm and 4.0 kg in the CP52 arm), and an assumed standard deviation of 6 kg. The statistical testing will be performed sequentially firstly by comparing CP arms with BI and then only if significant to then test for a difference between CP12 and CP52. Power is optimised by allocating more participants to the CP arms where the smaller difference is expected. With a sample of 1200 participants allocated as 200 BI, 500 CP12 and 500 CP52, we will have 99.95% power for the first test, to detect a difference of 2.3 kg between BI and combined CP and 92.87% power to detect a difference of 1.3 kg between CP12 and CP52. The total power of the study will be 92.82%.

### Clinical effectiveness

The primary analyses will assess differences in mean weight change from baseline to 12 months between the intervention groups. In order to investigate the impact of missing data, four analysis approaches will be taken: completers only, baseline observation carried forward (BOCF), last observation carried forward (LOCF) and a missing at random (MAR) analysis using a variance components model. For the LOCF, BOCF and completers analyses, fixed effect models for continuous normal data will be fitted to the 12 month weight data. The fixed effects will be intervention group, centre and baseline weight. For the MAR analysis, a model for multivariate normal data with the same fixed effects will be fitted using measured weights at each time point using generalised least squares. Coefficient estimates and their 95% confidence intervals will be calculated for each fixed effect.

All assumptions of the models will be checked using appropriate graphs (eq a Q-Q plot of residuals to check normality, residuals versus predicted values to check homogeneity of residual variance.) If the residuals are not normally distributed then the dependent variable may be transformed to normality, if there is no such transformation then non-parametric methods will be considered.

Secondary analyses will include analyses of weight change at 3 and 24 months; changes in blood pressure, waist circumference and fat mass at 3, 12 and 24 months; changes in biochemical measures at 12 months. These will be analysed using the same regression based models. Numbers of participants in each group achieving ≥5% and ≥10% weight loss at 12 and 24 months will also be explored.

Summary tables will be produced to look at the demographic distribution of the sample (age, sex, initial weight, BMI); attendance rates; time course of attendance; website usage.

### Cost effectiveness

#### Within-trial cost-effectiveness

The incremental cost-effectiveness ratio of the intervention is the main outcome of the economic evaluation and will be expressed as incremental costs per incremental change in weight/BMI for the within-trial evaluation. Cost items to be included will be the cost of the intervention (i.e. cost to NHS of referral packages and infrastructure related to the operation of the referral scheme), primary, secondary, and tertiary health care use associated with weight-related disease (especially diabetes, coronary heart disease, colon cancer, and musculo-skeletal disorders). At baseline, participants will complete a health care usage questionnaire covering health service attendances and any weight loss treatment for the previous 3 months. This questionnaire will be completed again at 3, 12 and 24 months. Analysis of uncertainty will be conducted with a non- parametric bootstrap of the sampled data to generate a cost-effectiveness acceptability curve showing the probability that the intervention is cost-effective at various willingness-to-pay thresholds per unit of outcome. The within-trial cost-effectiveness analysis will be conducted jointly with the outcome analysis in year 3 of the study. The data will also be incorporated into the economic model.

### Long term cost-effectiveness

Measuring cost-effectiveness in terms of costs per QALYs will allow the intervention to be compared with many alternative uses of existing NHS budgets. We will use the UK Health Forum’s “Obesity Micro-simulation Model”. The estimates the future burden of diseases by making evidence based extrapolations of selected risk factors specific to the following BMI related diseases; currently hypertension and stroke, diabetes mellitus type 2, cardiovascular diseases including angina pectoris, myocardial infarction, musculoskeletal disorders including osteoarthritis, low back pain and knee arthrosis; obesity associated cancers including colorectal, endometrial, ovarian, breast, cervical, prostate and possibly also gallbladder, pancreatic and renal. The micro-simulation incorporates a sophisticated economic module. The module employs Markov-type simulation of long-term health benefits, health care costs and cost-effectiveness of specified interventions. It synthesises and estimates evidence on cost-effectiveness analysis and cost-utility analysis within the countries. The model is used to project the differences in quality adjusted life years (QALYs), lifetime health-care costs and as a consequence of interventions incremental cost effectiveness ratios (ICERs). Sensitivity analysis is also done within this model. Outputs can be discounted for any specific discount rate.

### Qualitative analysis

Audio recordings will be transcribed verbatim by an external agency, checked for accuracy and imported into NVivo, along with the original audio files. Basic descriptive variables will be imported from the main trial database to analyse the interview and diary data. Initial analysis using a limited set of codes drawn directly from questions used in the topic guide will be conducted by at least two members of the team to ensure general reliability and appropriateness of categories. Analysis will then proceed iteratively in order to remain sensitive to the richness of data itself and develop a detailed hierarchy of emerging themes that address more implicit, and cross-cutting issues that emerge through the open- nature of the interviews. Exploiting the dynamic capacity of NVivo software, these themes will serve as the basis for comparison between participants. Analysis of the overall dataset will consequently enable both a narrative-based account of individual experiences, but also the extent to which they are intervention specific.

## Discussion

With one quarter of adults defined as clinically obese, and with growing financial pressures on health services, this trial will provide important information on the use of commercial providers to deliver weight management services in partnership with health professionals. Findings will provide transparent information about treatment and outcomes and will enable formation of clear guidance for commissioners and referring practitioners.

Guidance for commissioners from the Department of Health in England currently recommends 12 week interventions. While there is some evidence that longer interventions might improve weight loss, this evidence is inconsistent and generally comes from indirect comparisons between studies. Changing current practice to include longer referrals would require evidence of both greater clinical effectiveness and cost-effectiveness at a population level.

While the quantitative data in this study can provide guidance on the clinical and cost-effectiveness of the treatment, qualitative data will elucidate some key issues surrounding commercial partnerships, in particular patient perceptions regarding the acceptability of these interventions. This data will also provide insight into what participants perceive are the active ingredients of these interventions and what patients want weight management services to provide.

Data on psychosocial factors can be used to identify inter-individual differences in weight trajectories and could potentially be used to assist in stratifying patients to treatments likely to be effective. Data on changes in these factors during and following the intervention, and their association with weight trajectories, could potentially be used to inform improvements in existing interventions and the development of new interventions.

This trial endeavours to evaluate how effective this intervention would be in routine clinical practice, rather than under optimal controlled conditions. However, the conditions of this trial differ somewhat from those of routine clinical practice. Firstly, participants are recruited by letter and all participants who meet inclusion criteria and are invited. Thus our sample may be more heterogenous than those who a GP refers following a face-to-face consultation. Secondly, in two of the research centres, participants attend a research centre for their initial intervention allocation and all assessments. This enables greater control over data quality and participant follow-up, but differs from how the intervention would be rolled out in primary care.

Weight loss studies are notorious for high attrition, which can compromise the analysis and interpretation of data. While every effort will be made to enable participants to attend follow up assessments, continued participation in the trial is voluntary and we anticipate that there will be a substantial number of people who do not complete all measurements. However, this also reflects what would happen in clinical practice where many participants will not follow the programme they are referred to, or may not return for follow-up. Data will be analysed on an intention to treat basis. While no method of analysis is without limitations, this should give the best estimation of population level effectiveness.

### Research governance

#### Ethical approval

This is version 2.9 of the trial protocol dated 28^th^ July 2013. The Medical Research Council is the sponsor of the trial. This trial was registered at current controlled trials ISRCTN85485463 on 12^th^ October 2012. Ethical approval was received from NRES Committee East of England - Cambridge East (12/EE/0363) and local approvals from NRES Committee North West - Liverpool Central (12/NW/0678) and NRES Committee South Central – Oxford 12/SC/0508. Local NHS Research and Development approvals were received for all participating practices.

### Study sponsor

The Medical Research Council (MRC) will carry out the role of sponsor, with MRC Human Nutrition Research (HNR) the lead unit, in accordance with the Research Governance Framework and will take on responsibility for securing the arrangements to initiate, manage and finance (subject to funding) the study, and to ensure any risks are identified and managed and that the research is of high quality. MRC HNR has been certified since January 2006 to the quality management standard ISO9001:2008 by Lloyds Register QA and is subject to twice yearly external audit.

### Trial steering committee

The Trial Steering Committee (TSC) is chaired by Prof Martin Roland, Professor of Health Services Research in the University of Cambridge. Martin is Director of the National Primary Care Research and Development centre, Special Advisor to RAND Europe and has been a practising GP for over 30 years. Other independent members include: Prof Nick Finer, who is Honorary Professor at UCL and Consultant Endocrinologist at University College London Hospitals and one of the leading UK specialists in obesity management who has been a co-author in numerous obesity-related trials; Dr Judith Dawson, a full-time GP and Locality Lead for GP Commissioning in Northampton; and two patient/public representatives, Mrs Norma Scullion and Mr Graham Rhodes. Ms Polly Page, Director of Operations for MRC HNR and chair of the unit Research Review Board is also a member of the TSC.

The study is not blinded and carries low risk with no rules for early termination, so it is felt that it is neither necessary nor appropriate to have a specific Data Monitoring and Ethics Committee in addition to the TSC.

### Data handling and quality assurance

Participation will be under full informed consent, including for the storage and use of data collected. The Principal Investigator (PI) will be responsible for ensuring compliance with the Data Protection Act. Data collection forms will be kept in locked cabinets and an online database with secure encrypted transmission will be established by the database manager, accessible remotely by designated usernames and passwords and automatically backed up to ensure no loss of data. The PI and Trial Coordinator will monitor the accuracy of the database with validation checks against the data collection forms. All resulting datasets will be anonymised and stored securely.

### Research dissemination and data preservation for sharing

The investigators will analyse data according to pre-defined analysis plans in a timely manner. For those analyses described in this proposal this will be within the lifetime of the grant. The PI shall ensure that the results of the trial will be submitted for publication in a peer reviewed journal, regardless of the outcome. Authorship of publications will be determined by ICMJE guidelines. As project partners, Weight Watchers understand that they will have no influence on the data analyses or publications, but they will be able to see publications 14 days prior to submission to check any factual information relating to the company. All scientific papers and reports are peer reviewed by the HNR Research Review Board and signed off before publication. A lay summary of the research findings will also be sent to participants and participating primary care practices at the end of the study.

MRC HNR will be custodians of the data resulting from the study and will ensure compliance with the Data Protection Act and the MRC policy for data sharing and preservation. The HNR database manager will take responsibility for data curation and archiving and all data sets will be kept securely with no access from unauthorised personnel. Data will be stored so that it can be accessed, used and understood by subsequent users. When the investigators have completed their planned analyses, the anonymised data will be made available for use by others and will be shared under appropriate data sharing agreements. Primary data and the Trial Master File will be retained securely in their original form for a minimum of 10 years.

The commercial programme intervention will be delivered by an employee of company and the company will provide data on meeting attendance and website usage, but they will have no role in the study design, data analysis, data interpretation, or writing of the report.

### Trial status

Ongoing. Recruitment was completed in February 2014.

## Competing interests

ALA, SAJ, PA, and JCGH have received funding to their institutions from Weight Watchers and have given and received hospitality from providers of commercial weight loss services on a small number of occasions. PA and SAJ are conducting another publicly funded trial in which part of the intervention is delivered by and donated free by Slimming World and Rosemary Conley. Until January 2014, SAJ wrote a regular nutrition column for the Rosemary Conley Diet and Fitness magazine and received a fee. LC has received payment from Lighter Life Ltd for consultancy services. The other authors declare no conflicts of interest.

## Authors’ contributions

ALA is the Principal Investigator. ALA, SAJ, PA, JCGH, MS, and SC are grant holders. ALA and SAJ developed the research questions and designed the trial based on an original idea from SAJ. All authors contributed to the study design and development of the protocol. LC and AM provided statistical expertise in clinical trial design and AM will conduct the primary statistical analyses. MS and TM will lead the cost-effectiveness analyses outlined. SC will lead the qualitative workstream. AT is the Trial Coordinator. All authors have read and given final approval of the protocol.

## Pre-publication history

The pre-publication history for this paper can be accessed here:

http://www.biomedcentral.com/1471-2458/14/620/prepub
